# Interrater agreement between student and teacher assessments of endotracheal intubation skills in a self-directed simulation learning environment

**DOI:** 10.1186/s12909-023-04242-z

**Published:** 2023-04-18

**Authors:** Aida Mankute, Laima Juozapaviciene, Justinas Stucinskas, Zilvinas Dambrauskas, Paulius Dobozinskas, Elizabeth Sinz, David L. Rodgers, Evelina Pukenyte, Birute Kumpaitiene, Dinas Vaitkaitis

**Affiliations:** 1 Department of Emergency Medicine, Lithuanian University of Health Sciences, Kaunas, Lithuania; 2 Department ofAnaesthesiology, Lithuanian University of Health Sciences, Kaunas, Lithuania; 3 Department of Orthopaedics and Traumatology, Lithuanian University of Health Sciences, Kaunas, Lithuania; 4 Department of Surgery, Lithuanian University of Health Sciences, Kaunas, Lithuania; 5 Department of Disaster Medicine, Lithuanian University of Health Sciences, Kaunas, Lithuania; 6 Department of Anesthesiology and Perioperative Medicine, Medical Simulation Center, Penn State Health Milton S. Hershey Medical Center, Hershey, USA; 7 Interprofessional Simulation Center, Indiana University, Bloomington, USA; 8 Department of Infectious Diseases, Lithuanian University of Health Sciences, Kaunas, Lithuania; 9 Department of Cardiothoracic and Vascular Surgery, Lithuanian University of Health Sciences, Kaunas, Lithuania

**Keywords:** HybridLab, Self-directed learning, Skill assessment, Students, Teacher

## Abstract

**Background:**

Practical skill assessment is an important part of the learning process to confirm competencies in acquired medical knowledge.

**Objective:**

This study aimed to compare the assessments of endotracheal intubation skills using the HybridLab® methodology between students and teacher in terms of interobserver reliability.

**Methods:**

Reliability analysis was performed with observational data (data are reported according to STROBE guidelines). The study was conducted in two countries, the Lithuanian University of Health Science (LUHS) and Pennsylvania State University (PSU) in the US, between 1 January and 30 June 2020. A total of 92 students (60 from LUHS and 32 from PSU) were trained in endotracheal intubation using an algorithm-driven hybrid learning method. At the end of the training session, the participants had to complete the evaluation scenario, which was assessed by one of the students and evaluated remotely by a single teacher. The student assessment of the endotracheal intubation procedure was compared with the teacher’s assessment using correlation and estimation of the intraclass correlation coefficient.

**Results:**

Overall, the medians of the student and teacher assessments were both 100% (0%). Spearman’s correlation coefficient between the student and teacher assessments was 0.879 (*p* = 0.001). The intraclass correlation coefficient used for interobserver variations between the students and teacher was 0.883 (95% confidence interval from 0.824 to 0.923).

**Conclusions:**

The algorithm-driven hybrid learning method allows students to reliably assess endotracheal intubation skills to a level comparable with that of the teacher’s evaluation. This learning method has the potential to be a cost-effective and efficient way to provide high-quality education while also saving human resources.

## Background

The process of educating health care professionals involves great responsibility and students should be held to a high standard. Therefore, the evaluation process needs to include the assessment of teaching quality in addition to its accuracy and reliability [1]. However, great variability in the appraisal process, including tests, face-to-face examinations, and practical evaluations, may interfere with the efficacy of assessments. Few assessments involve high costs in terms of time or human and financial resources. Thus, these resources must be preserved without compromising the assessment quality. Furthermore, empowering adult learners to practice self-assessment in the learning process and building a culture of reflective analysis and peer assessment during training is an emerging area that has been attracting interest [2].

A hybrid learning method (HybridLab®) was developed and designed at the Lithuanian University of Health Science (LUHS) to efficiently combine e-learning and self-directed medical simulations. Hybrid simulation training combines online and in-person learning. In this learning method, participants have the opportunity to practice various skills in a simulated healthcare environment, including patient assessments, communication, decision-making, and teamwork. This method consists of a structured and standardised learning pathway, which includes studies on an e-learning platform, peer-to-peer hands-on training sessions in simulation classes using carefully elaborated learning algorithms, as well as peer assessment and direct feedback [3–6]. The learning method facilitates small-group peer-to-peer simulation training sessions with and without the direct supervision of the instructor. Training and evaluation do not require the direct participation of the instructor and can be set up at a preferred location for asynchronous video review. The main concept of this model is direct learning to attain certain specific medical competencies and provide learners with a toolkit that empowers them to work independently, adapt to skill training at their own personal learning pace, and build a culture of reflective analysis and peer assessment.

This hybrid model of directed and self-organised skill training is novel, and several published studies have documented its characteristics [3–6]. With the constantly expanding numbers of electronic learning and assessment tools, wider use of virtual reality, and video analysis of skill training (including artificial intelligence-driven systems), the field of medical simulations is evolving rapidly. There is an ongoing debate on how much medical simulation learning can be handed over to the learners themselves and how reliable the peer-to-peer assessment of clinical skills and competencies is. As the paradigm shifts, teachers are no longer considered the main participants in the teaching process, and students are becoming increasingly empowered and responsible for their learning. Although teachers remain the key assessors of the quality of learned skills and competencies for certification purposes, questions are raised on whether the students themselves could be involved in the competence and teaching evaluation processes and how reliable the peer assessment of acquired clinical skills is.

In this study, we compared the assessments of endotracheal intubation (ETI) skills taught by the HybridLab® methodology between students and teacher in terms of interobserver reliability. Our hypothesis is that the ETI skills taught by the HybridLab® methodology will be assessed similarly by students and teacher.

## Methods

This prospective cohort study was performed at two institutions, the Lithuanian University of Health Sciences (LUHS) and Pennsylvania State University (PSU), from 1 January to 30 June 2020. The study was approved by the Ethical Review boards of LUHS (permission no. BEC-MF-442) and PSU (permission no. HRP-591). All the participants provided written informed consent. Fifth-year medical students and first-year emergency medicine and intensive care residents taking their anaesthesiology rotation were included in this prospective cohort study, while those who had participated in similar practical skill training or had experience in ETI were excluded from the study. In total, 110 participants were included in this study; data from 92 students were used for the end analysis, while 18 participants did not complete the course and were excluded.

**Fig. 1:**
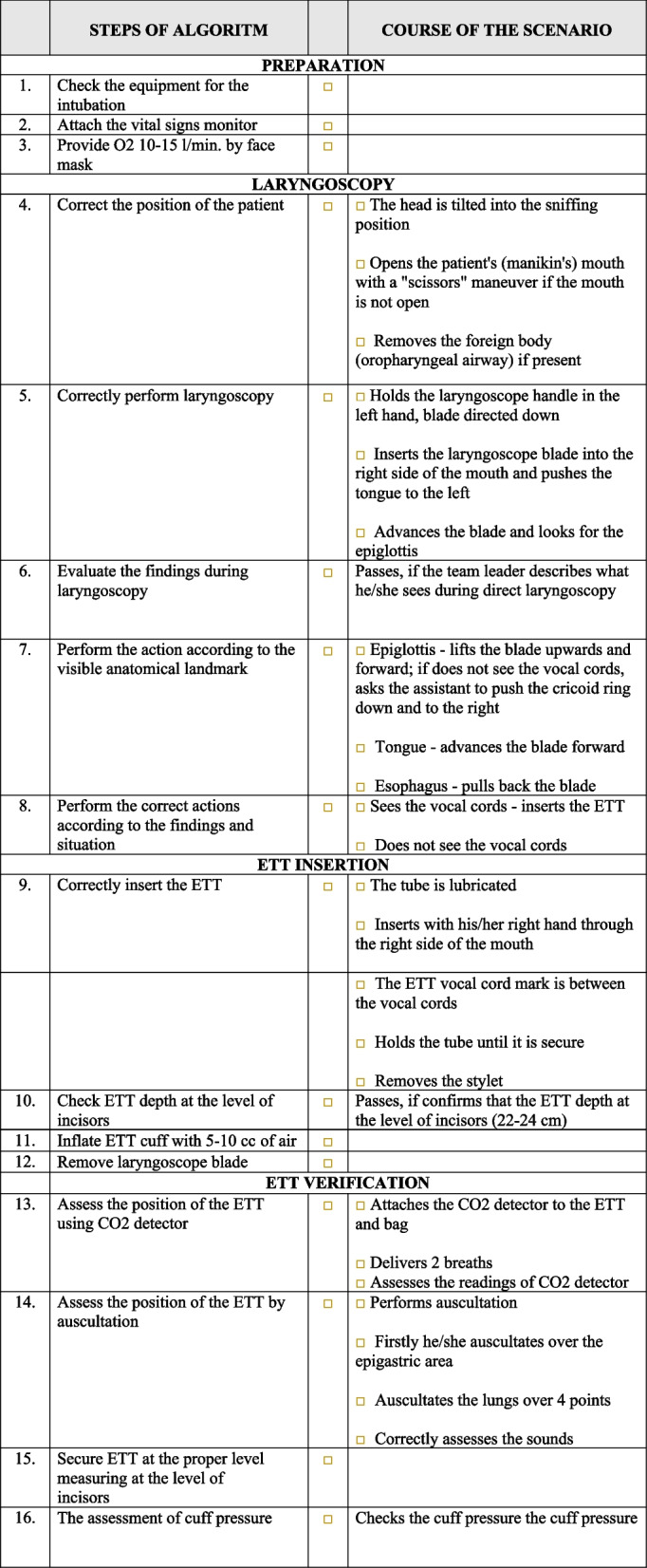
Checklist for clinical situation assessment and feedback

**Fig. 2:**
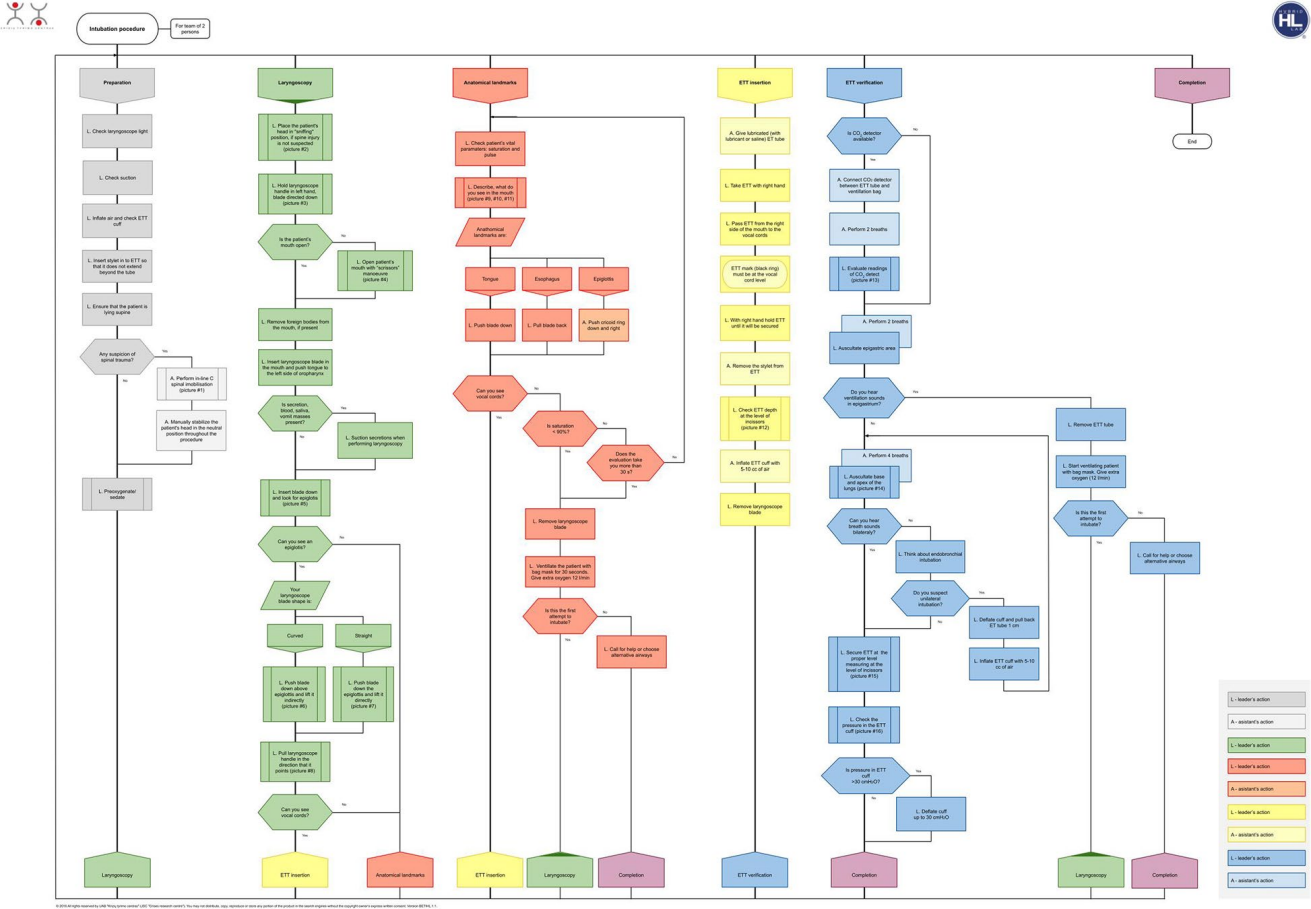
Algorithm intubation procedure

The participants studied the principles of safe airway management and practical skills for ETI using an algorithm-driven hybrid simulation learning method (HybridLab®, JSC Crisis Research Centre, Kaunas, Lithuania). The participants initially studied the necessary theoretical material, lectures, and algorithms in a virtual learning environment that included videos of each practical skill step. The students continued with practical training only after passing an online test comprising 10 questions. After the individual theoretical preparation, the participants organised themselves into groups of three with a role distribution, including a leader, an assistant, and an assessor for peer-to-peer practical skills training sessions in the HybridLab® classrooms. Participants in each group belonged to the same level of education (students or residents). The team members had to switch roles by applying the learning-training-teaching principle. The preplanned estimated duration of skill training was 3 h. While learning practical skills, the participants used the manikin and required ETI equipment and had the supplementary support of handheld tablets containing proprietary educational software, interactive algorithms, electronic scenarios, videos, and checklists for clinical situation assessment and feedback (Fig. 1) as well as learning algorithms (Fig. 2), which enabled simulation and training in the absence of a technician or instructor. During the training session each participant had to perform 4 learning scenarios. At the end of the training session, the participants completed the evaluation scenario. Each evaluation scenario had to be assessed by one of the three students during the session. Training sessions were video recorded using cameras installed in the lab. The recordings of both the training and evaluation scenarios were viewed and evaluated remotely by a single teacher (LUHS). Standardised checklists were used for evaluation, which were the same as those used in the training class. The evaluation and feedback from the teacher were provided to the students via email.

Completion of all ETI steps (16 actions) according to the checklist was evaluated with a sum of 100%. The assessments of the ETI procedure by students were compared with those by the teacher. The assessment results were divided into groups as per the scores: 100%, 75–99%, and 0–74%. Matching and nonmatching assessments between students and teacher were registered. In addition, the effect of the participants’ sex and experience level (student/resident) in comparison to the teacher’s assessment was evaluated. Additionally, differences in teacher assessments between countries were analysed.

### Statistics

The primary effect variable used for the statistical power calculation was the difference in assessments between the students and teacher. With the assumption of a difference in means of 5%, standard deviation of 10%, power of 0.8, and risk of 0.05 for type-1 error, 64 study participants were needed.

A Kolmogorov–Smirnov normality test was used to determine whether the data were normally distributed. As the data were not normally distributed, they are presented as medians (interquartile ranges) and rates. Spearman correlation and Bland–Altman analyses were used to calculate the correlation between student and teacher assessments. Interrater reliability analysis was performed using one-way random intraclass correlation coefficients (ICCs). Values less than 0.5 indicated poor reliability, between 0.5 and 0.75 indicated moderate reliability, between 0.75 and 0.9 indicated good reliability, and greater than 0.90 indicated excellent reliability [7]. Additionally, a nonparametric Mann–Whitney U test for independent samples was used. Statistical significance was set at *P* < 0.05. SPSS software (SPSS, Chicago, Ill., USA) was used for all calculations.

## Results

Data on the demographic characteristics of the participants (*n* = 92) are presented in Table 1. The results of assessments completed independently by the students and teacher for each of the individual ETI steps (16 actions) are presented in Table 2.

**Table 1:** Participants’ demographic data (LUHS/PSU)

Demographic characteristics	Median (range) or N (%)
Age (years)	23 (21 to 24)
Sex (female/male)	59/33 (64/36%)
Level of education (student/resident)	80/12 (87/13%)
Country (LUHS/PSU)	60/32 (65/35%)

*LUHS* Lithuanian university of health science, and *PSU* Pennsylvanian State University (USA)

**Table 2:** Assessment of completion of the 16 steps of endotracheal intubation: comparison between student and teacher

Action	Student evaluation	Instructor evaluation
1. Checks equipment	97%	96%
2. Connects monitor	92%	91%
3. Preoxygenates correctly	91%	89%
4. Ensures correct patient position	97%	97%
5. Correct laryngoscopy	100%	98%
6. Describes laryngoscopy findings	98%	98%
7. Performs the right actions according to visual anatomical findings	100%	98%
8. Performs the right actions according to findings and situation	100%	100%
9. Correct insertion of the ETT	100%	100%
10. Checks ETT depth	99%	100%
11. Inflates ETT cuff	100%	100%
12. Removes laryngoscope blade	100%	100%
13. Asks for CO_2_ detector and checks it	99%	98%
14. Auscultates chest to assess position of ETT	100%	100%
15. Secures ETT	95%	97%
16. Assess ETT cuff pressure	100%	100%

*ETT* Endotracheal tube

**Fig. 3:**
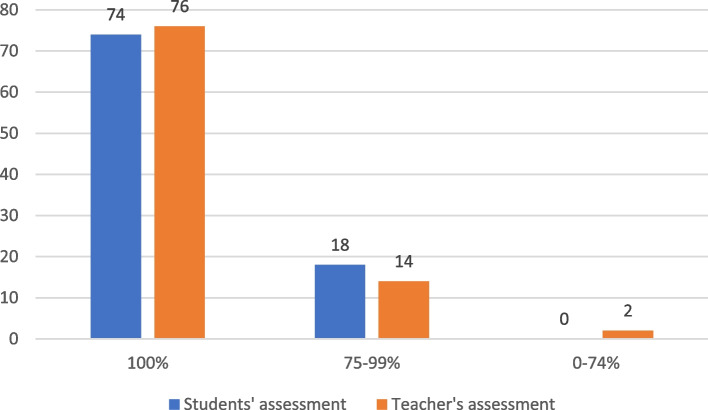
Distribution of 100%, 75–99%, and 0–74% assessment results between the students and teacher

No statistically significant difference was reported between the student and teacher evaluations, with an overall median score of 100% (100 to 100%) in both groups (p = 0.793, Mann–Whitney U test). The distribution of 100%, 75–99%, and 0–74% assessment results between the students and teacher are presented in Fig. 3.

**Fig. 4:**
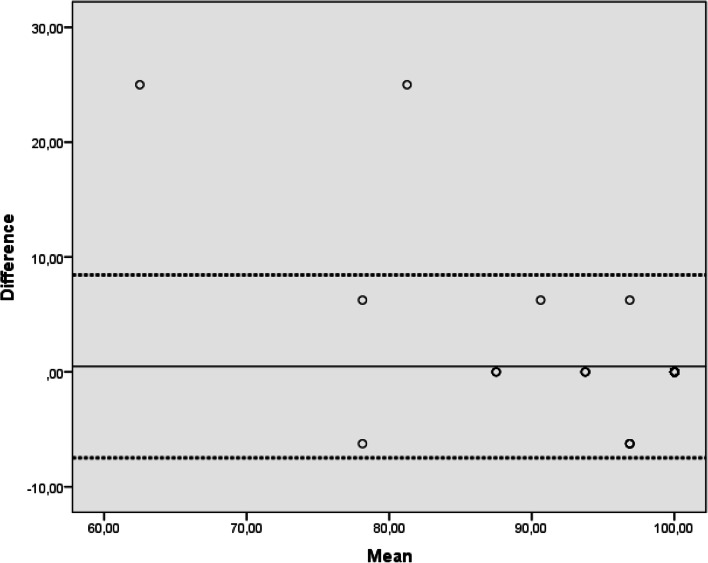
Bland–Altman analysis for the agreement between students’ and teacher’s assessments

Spearman’s correlation coefficient between student and teacher assessments was 0.879 (*p* = 0.001). The Bland–Altman graph is presented in Fig. 4.

The ICC used for the analysis of interobserver reliability between the students and teacher showed good agreement between the two groups of assessors, with a coefficient of 0.883 (95% confidence interval between 0.824 and 0.923).

Of the 92 assessments, nine did not match between the students and the teacher (94% [78 to 94%] and 88% [72 to 100%], respectively; *p* = 0.796, Mann–Whitney U test). Of these nine assessments, five student evaluations were higher than the teacher’s assessments but not statistically significant (94% [78 to 97%] and 75% [59 to 91%], respectively, *p* = 0.151, Mann–Whitney U test).

Participants’ sex and education level (student/resident) had no effect on the teacher’s assessments (*p* = 0.092 and 0.283, respectively, Mann–Whitney U test). However, when comparing the teacher’s assessments between countries, Lithuanian students scored significantly higher than American students (100% (100 to 100%) vs. 100% (94 to 100%), *p* = 0.01, Mann–Whitney U test).

**Fig. 5:**
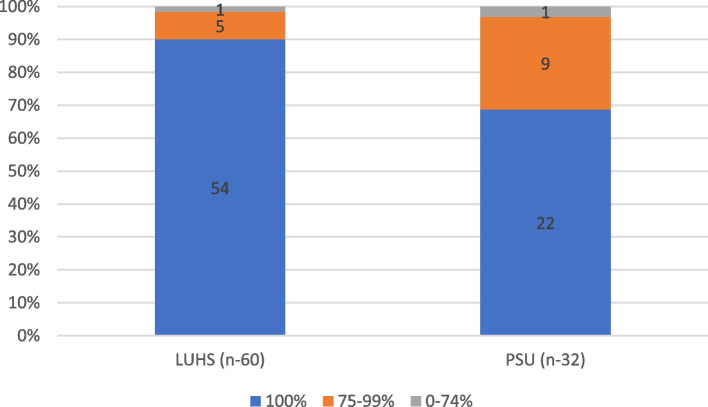
Distribution of 100%, 75–99%, and 0–74% assessment results between LUHS and PSU. LUHS, Lithuanian University of Health Science and United States of America; PSU, Pennsylvanian State University

The distributions of assessment scores across the three categories 100%, 75–99%, and 0–74% between LUHS and PSU are presented in Fig. 5.

## Discussion

This study demonstrated that students can reliably assess ETI skills learned in simulation-based hybrid training sessions when they are provided with a structured skill assessment tool and an appropriate video tutorial. A high positive correlation [8] was observed between the assessments provided by the students and teacher (*r* = 0.879, *p* = 0.001). Additionally, the ICC for interobserver variations between the student and teacher assessments was 0.883, and the 95% confidence interval ranged from 0.824 to 0.923, which can be regarded as “good” to “excellent” [7, 9]. This is in concordance with the findings of a systemic review by Yu et al. [10], which showed that peer teaching of medical students achieves learner outcomes comparable to those through conventional faculty-led teaching in highly selective contexts. This result could be explained by the fact that peer teachers (students) may have an enhanced motivation to learn the material that they teach, and this motivation is known to be related to learning [10, 11]. In addition, peer teaching results in a deeper processing of information, which increases conceptual learning [10], suggesting that for students participating in practical skills, peer-to-peer learning can be reliably assessed by their peers as well as by faculty teachers. Clear and structured algorithms, well-defined rules for formative and summative assessment, and the ability to learn at their own pace are the key factors ensuring the functioning and reliability of the self-directed learning HybridLab® platform [6, 12].

The success rate of the students in achieving the predefined learning outcomes for the ETI skill set in this study was very high, with an overall median score of 100% (students were encouraged to practice until they reached full automaticity in performing the task). This is in accordance with the results of another study, which reported an overall average of 96% among medical students learning neonatal resuscitation skills [4]. HybridLab® training, as an interactive algorithm-driven approach in a stepwise manner, does not allow students to miss a single step during their training and assessment. Additionally, we believe that the awareness of being recorded during the study process in the HybridLab® class leads to honest and objective student performance. Therefore, the positive learning outcomes achieved using the hybrid training method help improve medical education. Furthermore, a recent randomised study showed that students in the HybridLab® training group demonstrated better clinical application of the acquired skills and better adherence to patient safety procedures during testing in a clinical environment with real patients than that observed in a standard simulation-based learning group [6].

Our results revealed that students’ sex and education level (student/resident) had no effect on the teacher’s assessment; however, the teacher’s assessment scores were significantly higher for Lithuanian students than for American students. Although the teacher’s assessment medians differed significantly, we believe that the difference is not practically significant, as the medians for both countries were 100%. These small differences could be explained by the fact the American students were encountering this training method with the HybridLab® platform for the first time, while the Lithuanian students were familiar with this system prior to the study and had used it while learning other practical skills. Additionally, certain institutional and/or cultural differences are possible in the interpretation of certain components of corresponding skills. This indicates that even with a rather robust assessment model (when structured skill assessment forms and video tutorials are used), the faculty needs to be trained and briefed on how the evaluation should be carried out, especially in high-stakes certification or examination settings. However, the results also suggest that this training method can be successfully adopted for standardised medical training in different institutions and countries.

The main strengths of the study are that it was carried out in an international and multicentre fashion, and the participant number included in the study was sufficient based on statistical power estimates to support the primary outcomes of the study. The main limitation of our study is that we could not prove that the students’ peer-to-peer assessment could be used not only during formative but also in summative high-stakes assessments in medical education. The current results clearly suggest that the use of the same checklist and instructional videos led to comparable assessment results between the students and teachers. However, with other scenarios, for example, if students knew that there would be no further skill evaluation by the teachers, the results may differ. Although we suggest that the participation of faculty in evaluation is still needed [12–14], their involvement could be reserved for situations where there is a need to certify a certain level of competence or skills and/or perform high-stakes summative assessment, while the majority of low-stakes formative assessments could be handed to peer learners [14, 15]. To stimulate and document the progress of the students, faculty members could randomly evaluate some of the selected scenarios/skills in a group of students if several practical skills are taught using the self-organised learning methodology during a module/semester of the academic year. If students are aware that any situation could be randomly selected for instructor assessment, they would make equal efforts to complete all assigned tasks and perform well. Moreover, this method allows for a more efficient use of available human resources in the teaching process with less disruption to clinical work because more instructor tasks can be performed asynchronously online. Although we did not perform a cost-effectiveness analysis in this study, we believe that this training method using the HybridLab® platform, mobile technologies, and algorithm-driven learning offers an opportunity to reduce the cost in time and human resources, as well as creates unique possibilities for learners to explore the benefits of autonomous, self-regulated learning and develop new feedback and peer assessment techniques.

We conclude that the algorithm-driven hybrid learning method allows students to reliably assess ETI skills when compared with teacher evaluations, thus allowing the effective use of peer-to-peer formative assessments of simulation-based skills training using the directed self-organised learning model. Our recent data from the randomised study showed that learning in the HybridLab® group translates into better clinical application of the acquired skills and better adherence to the patient safety procedures during testing in the clinical environment with real patients in comparison to the standard simulation-based learning group. This learning method has the potential to be a cost-effective and efficient way to provide high-quality education while also saving human resources.

## Data Availability

All data generated and analysed during the current study are available from the corresponding author on reasonable request.

## Abbreviations

LUHS: Lithuanian university of health science
PSU: Pennsylvania state university
ETI: Endotracheal intubation
USA: United States of America
ICC: Intraclass correlation coefficient
